# Avoiding the Pitfall: Ectopic Pelvic Kidney Mimicking a Pelvic Mass in a Patient With Recurrent Urinary Tract Infections. A Case Report

**DOI:** 10.1002/ccr3.72284

**Published:** 2026-03-13

**Authors:** Christian Damien Tchuisseu Ngapjang, Vanina Wanko, Christine Shaw, Denis Georges Teuwafeu, Ronald Gobina, Larisse Usongo Nchang, Emmanuella Manka'a, Verla Vincent Siysi

**Affiliations:** ^1^ Department of Internal Medicine and Pediatrics, Faculty of Health Sciences University of Buea Buea Cameroon; ^2^ Douala General Hospital Douala Cameroon; ^3^ St Vincent Clinical Faculty University of Alabama Alabama USA; ^4^ Buea Regional Hospital Buea Cameroon

**Keywords:** abdominopelvic CT scan, congenital renal anomaly, ectopic kidney, pelvic kidney, pelvic mass, recurrent urinary tract infection (UTI)

## Abstract

Recurrent urinary tract infections (UTIs) are a common clinical presentation, but they can sometimes be the key signpost to a hidden anatomical surprise. We report the case of a 55‐year‐old obese woman with a decade‐long history of recurrent UTIs and pelvic fullness. Examination revealed a vague pelvic mass, suspicious for a gynecological neoplasm. A contrast‐enhanced computed tomography (CT) scan definitively diagnosed an asymptomatic ectopic pelvic kidney, with an empty right renal fossa. The pelvic kidney was the likely anatomical predisposing factor for her recurrent UTIs, which were complicated by antibiotic resistance due to prior self‐medication. The case underscores the critical importance of including renal ectopia in the differential diagnosis of a fixed pelvic mass, particularly in patients with recurrent UTIs, to prevent misdiagnosis and iatrogenic injury, and highlights cross‐sectional imaging as the essential diagnostic tool.

## Key Clinical Message

Consider a congenital pelvic kidney in the differential diagnosis of a pelvic mass. Incidental discovery on cross‐sectional imaging provides a definitive diagnosis, prevents unnecessary interventions, and identifies the hidden cause of recurrent urinary tract infections (UTIs), guiding appropriate long‐term management.

## Graphical abstract

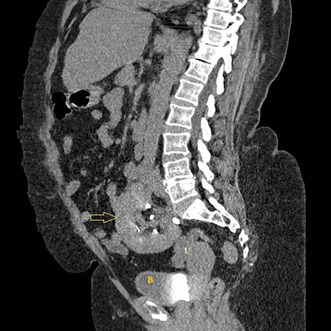

A 55‐year‐old woman with recurrent UTIs and a fixed pelvic mass on examination was intially suspected of having a neoplasm. Contrast‐enhanced CT revealed an ectopic pelvic kidney (**yellow arrow**, adjacent to uterus U), confirming an asymptomatic congenital anomaly as the cause. Imaging is essential to avoid misdiagnosis and iatrogenic injury.

## 1 Introduction

A pelvic kidney is a rare congenital anomaly, with an incidence between 1 in 2200 and 1 in 3000 autopsies [1]. It results from the failure of the metanephros to ascend from the pelvis to the lumbar region during weeks 6–9 of embryogenesis. While often asymptomatic and discovered incidentally, an ectopic pelvic kidney is a clinically significant finding. Its abnormal position and potential for aberrant vasculature or ureteric drainage predispose patients to complications such as vesicoureteral reflux, ureteropelvic junction obstruction, nephrolithiasis, and recurrent urinary tract infections (UTIs) [2]. This condition can be potentially mistaken for a pelvic tumor, leading to unnecessary interventions or to be injured during abdominal surgery if unrecognized [3]. We present a case that underscores the importance of considering this anatomical variant in the differential diagnosis of a pelvic mass or etiology of recurrent UTIs.

## 2 Case Presentation

A 55‐year‐old obese, nulliparous woman presented to the emergency department with a 1‐week history of progressive asthenia, cough, low‐grade fever (37.8°C), and dysuria. The patient reported a 10‐year history of recurrent UTIs for which she intermittently self‐medicated with antibiotics (typically amoxicillin + clavulanic acid). She also described a persistent, vague sensation of pelvic fullness and distension for several years, which she attributed to her obesity.

On admission, vital signs were remarkable for mild tachycardia (102 beats/min). The physical examination noted android obesity, which was a challenge for a good abdominal examination. While deep palpation in the hypogastrium elicited a sense of a firm, deep‐seated structure, a definitive, discrete mass could not be reliably delineated due to overlying adiposity and bowel. A bimanual pelvic examination, however, revealed a firm fixed fullness in the posterior pelvis, distinct from the uterus and adnexa, raising initial clinical suspicion for a pelvic neoplasm such as an ovarian mass or complex fibroid. The urine was turbid and the rest of the physical examination was unremarkable. Initial differentials included recurrent UTI, pelvic mass of uncertain etiology, and atypical pneumonia.

## 3 Investigations

Initial investigations were directed toward the working diagnoses. Urine culture subsequently grew *Escherichia coli* (> 10^5^ CFU/mL) sensitive to Nitrofurantoin, Ciprofloxacin, Amikacin, Meropenem, intermediate to Ceftriaxone, resistant to Amoxicillin + Clavulanic acid and Ampicillin.

**FIGURE 1:**
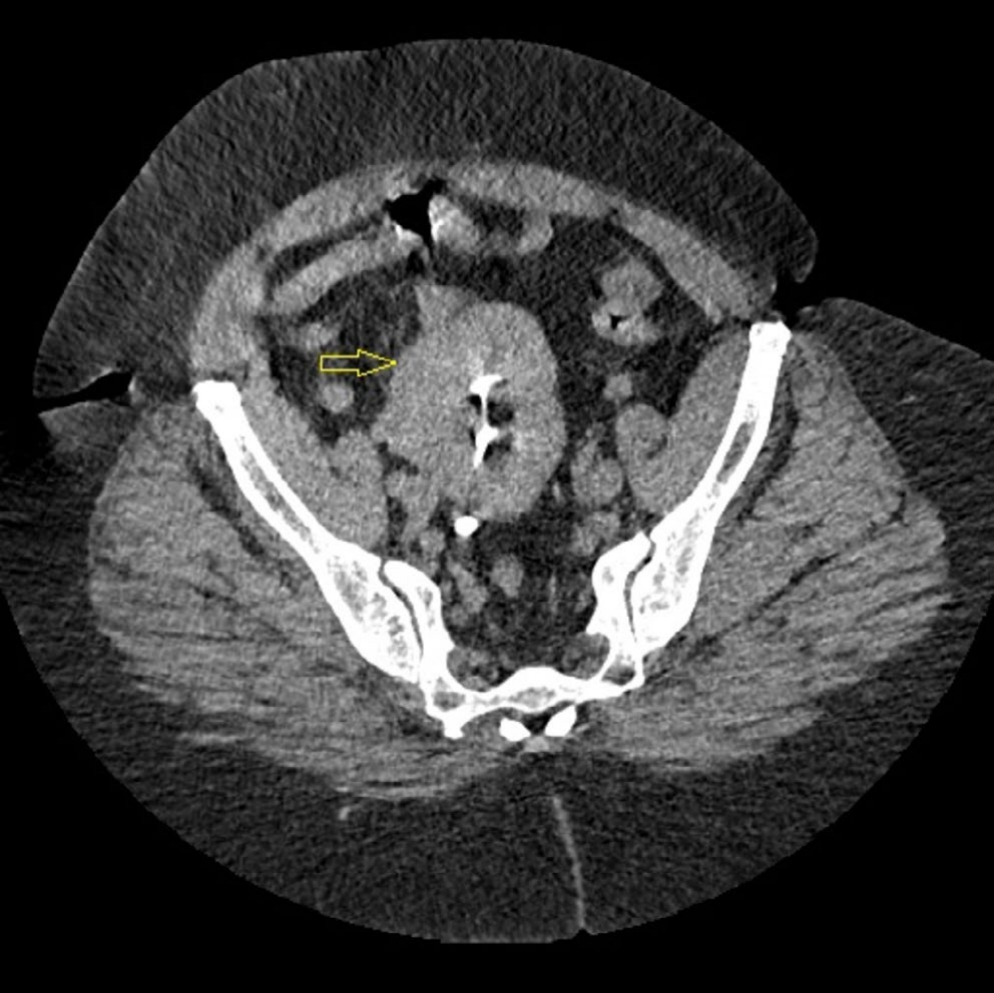
Axial view, contrast‐enhanced CT scan. The image shows the ectopic pelvic kidney (yellow arrow) located in the pelvis, between the two iliac crests.

**FIGURE 2:**
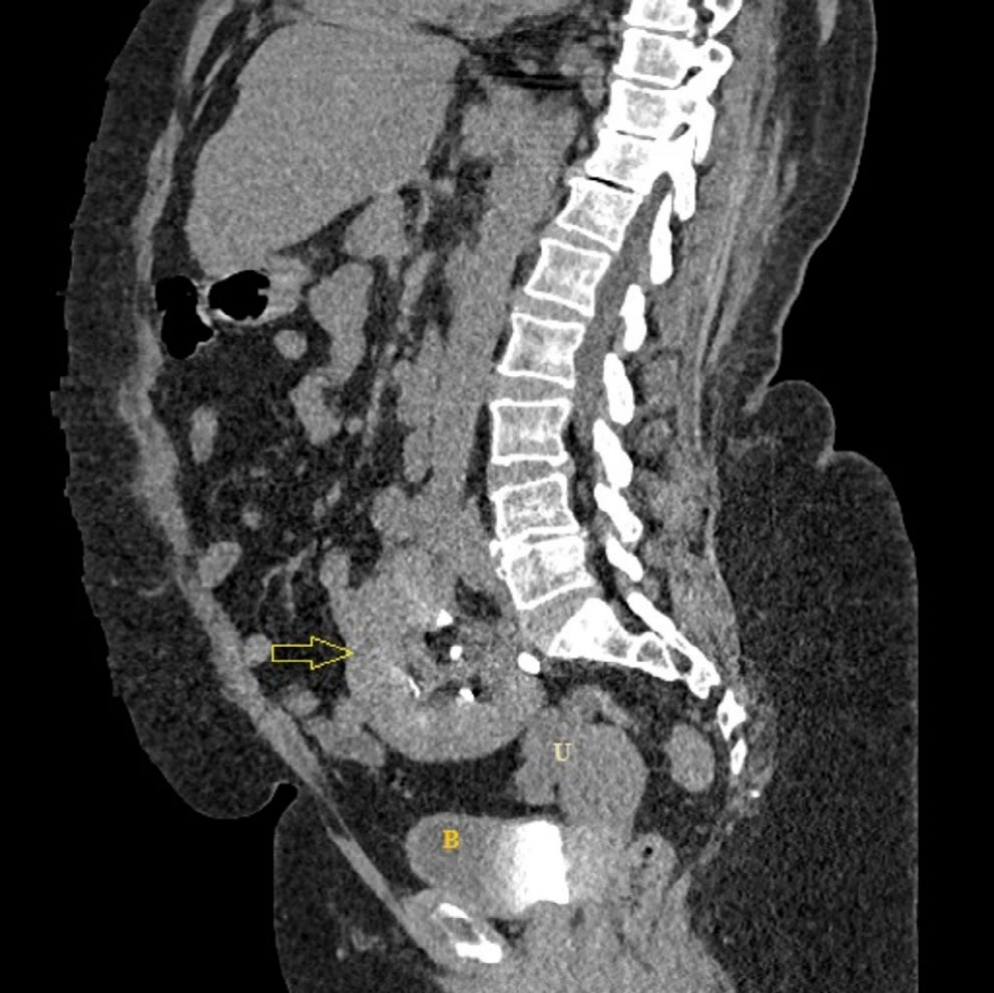
Sagittal view, contrast‐enhanced CT scan. This reconstruction demonstrates the ectopic kidney (yellow arrow) in the pelvis, at the level of L5–S1, having close contact with the uterus (U).

**FIGURE 3:**
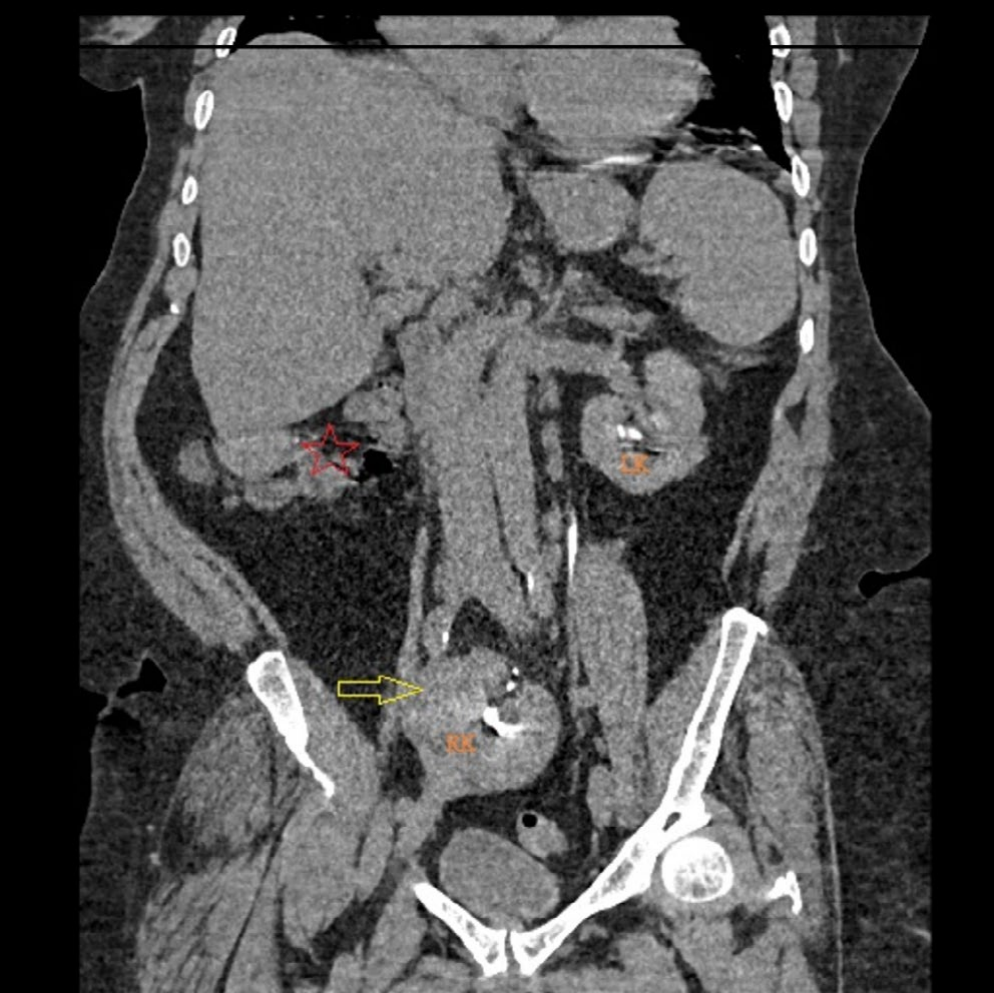
Coronal view, contrast‐enhanced CT scan. This reconstruction demonstrates the ectopic kidney (yellow arrow) in the pelvis and the empty right renal fossa (red star). The left kidney (LK) is in its normal anatomical position.

A contrast‐enhanced thoraco‐abdomino‐pelvic computed tomography (CT) scan was performed to rule out pneumonia and characterize the pelvic mass. The scan revealed no evidence of pneumonia or pelvic tumor. However, it revealed a striking anatomical variant: an empty right renal fossa and a normally sized and functioning ectopic kidney located in the pelvis, lying posterior to the urinary bladder and anterior to the sacrum (Figures 1, 2, 3). That kidney showed normal contrast excretion and no signs of hydronephrosis or calculi. The ureter was short and inserted normally into the bladder. The left kidney was orthotopic and normal. The final diagnosis was recurrent *E. coli* UTI complicating a right ectopic pelvic kidney.

## 4 Treatment

The patient received empirical intravenous Ciprofloxacin (400 mg/12 h), which was continued until Day 10 after confirmation of sensitivity for the isolated germ in urine culture. The symptoms (dysuria, fever, asthenia) fully resolved within 5 days. No intervention was required for the ectopic kidney itself.

## 5 Outcome and Follow‐Up

The patient was counseled extensively on her condition. Given the increased lifetime risk of complications, a structured follow‐up plan was instituted. After completing the 10 days treatment, she was referred to a urologist outpatient consultation for long‐term surveillance, which includes annual monitoring of blood pressure, serum creatinine, and urinalysis, with a renal ultrasound two times a year to assess for hydronephrosis or calculi formation. She was emphatically advised to inform all future healthcare providers, especially surgeons, of her renal anatomy prior to any abdominal or pelvic procedure.

## 6 Discussion

This case illustrates a classic diagnosis pitfall where a congenital anomaly mimics a pathological mass. The presentation (recurrent UTIs, intermittent pelvic discomfort and a fixed pelvic mass in a nulliparous woman) was highly suggestive of a gynecological neoplasm (uterine fibroids, ovarian mass), resolved definitively by imaging.

The pathophysiology linking pelvic kidney to recurrent UTIs is well‐documented [1, 4]. The ectopic position often results in abnormal ureteral insertion, malrotation, or aberrant vasculature, predisposing to functional obstruction, urinary stasis, and vesicoureteral reflux, all risk factors for recurrent infections [5, 6, 7]. Our patient's long history of UTIs aligns with this being a common presenting feature of an otherwise silent condition, as highlighted in similar case reports [4, 8]. This reinforces that an ectopic pelvic kidney must remain a fundamental consideration in the differential for any fixed pelvic fullness or presumed mass, particularly in a patient with a congruent history of UTIs or nephrolithiasis [9]. In such scenarios, modern cross‐sectional imaging, such as the CT scan used in our case, serves as the definitive diagnostic tool [10]. It safely navigates this diagnostic challenge by confirming the anatomical variant, ruling out pathology, and directly informing subsequent management.

Case reports of pelvic kidneys are well‐documented in the literature [11, 12], and the primary importance of their identification lies in avoiding misdiagnosis as a pelvic tumor and in preprocedural planning to prevent iatrogenic injury during abdominal or pelvic surgery [3]. The management of an asymptomatic pelvic kidney is conservative but requires regular monitoring for potential complications. This involves regular monitoring of renal function, screening for hypertension (which is more common in renal anomalies), and periodic imaging to detect obstruction or calculi [3, 13]. Our instituted follow‐up plan reflects this standard. A symptomatic patient may need surgery in case of obstruction or malfunctioning [3].

Recurrent UTIs defined as ≥ 2 acute UTIs within 6 months or at least 3 within a year are strongly influenced by anatomical defects like ectopic pelvic kidney [14]. The most common germ in cause is *E. coli* and the treatment usually includes Amoxicillin + Clavulanic acid [14, 15, 16]. The patient's decade‐long history of recurrent UTIs managed with intermittent self‐medication using Amoxicillin + Clavulanic acid is not a minor detail; it is a central, modifiable risk factor that directly explains the specific resistance pattern observed in her *E. coli* isolate. This practice illustrates a powerful selective pressure that shaped her personal microbiome and compromised future treatment options. The use of Ciprofloxacin as first intention in this context was a good empirical choice, as illustrated in many studies [4, 17]. Referring the patient to the urologist was very important in order to guarantee the patient's further management strategies including antibioprophylaxis and counseling [4, 14].

## 7 Conclusion

Ectopic pelvic kidney is a classic great mimicker: a congenital anomaly that should be considered in cases of recurrent UTIs or the differential diagnosis of a pelvic mass. Timely cross‐sectional imaging is essential for definitive diagnosis, preventing misdiagnosis, guiding surveillance, and avoiding iatrogenic harm.

## Author Contributions

**Emmanuella Manka'a:** visualization. **Verla Vincent Siysi:** supervision, validation. **Vanina Wanko:** writing – review and editing. **Larisse Usongo Nchang:** visualization, writing – review and editing. **Christine Shaw:** writing – review and editing. **Ronald Gobina:** project administration, visualization. **Denis Georges Teuwafeu:** visualization. **Christian Damien Tchuisseu Ngapjang:** conceptualization, data curation, methodology, resources, writing – original draft, writing – review and editing.

## Funding

The authors have nothing to report.

## Ethics Statement

Written informed consent was obtained from the patient.

## Conflicts of Interest

The authors declare no conflicts of interest.

## Data Availability

Data openly available in a public repository that issues datasets with DOIs.
